# LETTER TO THE EDITOR BY MEGAN HALL

**DOI:** 10.2174/1389202911314020001

**Published:** 2013-04

**Authors:** Jennifer Saucier, Katrina Merrion, Janine Mash, Barbara Pettersen, Megan Hall, Zachary Demko

**Affiliations:** Natera Inc

Sir,

We read with interest the recent review by Santiago Munné entitled, “Preimplantation Genetic Diagnosis for Aneuploidy and Translocations Using Array Comparative Genomic Hybridization” (1). As part of the review of array comparative genome hybridization (aCGH), the author provides additional information on other 24-chromosome preimplantation genetic diagnosis/screening (PGD/PGS) techniques. As a commercial lab that offers single nucleotide polymorphism (SNP) microarray analysis for PGD/PGS, we would like to comment on a few claims that were made within this article regarding SNP microarrays. 

(Table **1**) in the article summarizes the differences between 24-chromosome PGD/PGS techniques. This table contains a number of errors regarding SNP microarray detection capabilities as it groups the different approaches of SNP microarray analysis under a single heading of “SNPs”. SNP technologies that employ a combination of qualitative and quantitative data analysis detect far more abnormalities than those that use just one type of analysis. First, it is not accurate to say that “SNPs” cannot detect tetraploidy. SNP microarrays using qualitative/quantitative analysis can detect some forms of tetraploidy; this method will not detect 2:2 tetraploidy, though is indeed capable of detecting 3:1 tetraploidy. Second, SNP microarray technologies that use a qualitative/quantitative approach can detect meiotic and mitotic duplications without recombination (3); the table incorrectly states that SNP microarray approaches cannot detect these abnormalities. Lastly, it is an exaggeration to state that aCGH approaches are able to detect all unbalanced translocations and SNP microarray approaches can only detect some; both approaches are equally limited in their inability to detect very small deletions and duplications (both have a similar threshold in that they typically detect DNA segments greater than 6Mb). 

We also question a number of statements the author makes about aCGH and SNP microarray within the body of the paper. The author acknowledges that aCGH cannot detect haploidy or polyploidy but claims that this is a small limitation, as the majority of the haploid or polyploid embryos tested (7.7%) had additional detectable abnormalities; however, these additional abnormalities are not named. It is our experience that other abnormalities are not typically found with 69,XXX. Next, the author credits SNP microarray with the ability to detect uniparental disomy (UPD) but then goes on to use the incidence of UPD of chromosome 15 (UPD-15) to say that UPD in general is a very rare event. Chromosome15 is only one of six imprinted chromosomes (6, 7, 11, 14, 15, and 16), which if UPD is present, could lead to the birth of a baby with a severe genetic syndrome (4). We feel that detection of UPD prior to embryo transfer decisions is highly beneficial to, and desired by, couples undergoing IVF with PGD/PGS. Furthermore, in regards to PGD/PGS for reciprocal and Robertsonian translocations, we would like to clarify that not all SNP microarray approaches can differentiate between normal and balanced (carrier) embryos. Last, the author correctly points out that SNP microarray approaches require parental DNA analysis prior to the embryo sample analysis. However, our lab does not charge a cancellation fee for this parental analysis when IVF cycles are cancelled, thus patients do not pay for unnecessary parental testing.

We appreciate that the author mentions SNP microarray analysis that employs a qualitative/quantitative approach will avoid many of the limitations inherent to the purely qualitative or quantitative approaches. However, the review references our article (Johnson DS *et al.* [2], reference 77 in the original paper) as an aCGH technology, when in fact we utilize a SNP-based approach with bioinformatics analysis. Natera’s PGD/PGS Parental Support^TM^ method utilizes SNP measurements of parental and embryonic samples, giving us the ability to analyze both qualitative and quantitative data from each chromosome to determine the embryonic chromosomal copy number. In addition, the Parental Support^TM^ method utilizes a much more sophisticated bioinformatics-based analysis (2) than the combined approach described in the article. 

New methods should always be validated against more established ones, but given the errors rates reported with FISH PGD/PGS (as stated by the author at the beginning of the paper), we strongly disagree that validation studies using FISH for reanalysis should be considered the gold standard (2, 5). Natera thus supports the creation of an oversight body to administer proficiency testing for laboratories offering 24-chromosome PGD/PGS.

Sincerely,
