# Supplementary material for: LETTER TO THE EDITOR BY MEGAN HALL
Source: Curr Genomics. 2013 Apr;14(2):79. doi: 10.2174/1389202911314020001 (PMC3637680; doi:10.2174/1389202911314020001)
Supplement: Related texts

# LETTER TO THE EDITOR BY SANTIAGO MUNNÉ

**Published:** 2013-04

**Authors:** Santiago Munné

**Affiliations:** Reprogenetics

Sir,

We stand by our paper and the previous one by Bisignano *et al.* (2011) but would like to add a few clarifications.

Depending on the density of the array used, aCGH has a resolution between 6Mb and 1Mb. The paper of Fiorentino *et al.* (2011) in Fig. (**3**) illustrates an abnormal embryo from a 46,XY,t(3;18)(p26.3;q12) translocation with a 2.5M deletion in chromosome 3 (3p26.3-pter). Furthermore, any unbalanced translocation can be detected if three of the four chromosome arms are detected, therefore for a translocation to be missed with the high-resolution aCGH chip, it will require two fragments to be below 1Mb. Indeed of 926 procedures of PGD for translocations previously analyzed by FISH, none had two such small fragments (Colls *et al.* 2012), although a few have been described recently. 

About polyploidy, it is well known from the times of FISH (Munne *et al.* 1994) that the majority of monospermic polyploid embryos are chaotic mosaics. For the present paper, we retrospectively reviewed the data on 91,073 embryos analyzed by FISH. Of those 7.7% were polyploid or haploid but the vast majority were again complex chaotic abnormalities, for example 2X2Y, 6[13], 4[14], 2[15], 2[16], 4[17], 6[18], 4[19], 4[20], 4[21], 4[22]. Others were a combination of polyploidy and aneuploidy, and the vast majority of haploid embryos were hapohaploid i.e. XO, 0[13], 1[14], 2[15], 0[16], 0[17], 1[18], 1[19], 0[20], 0[21], 1[22]. The theory advanced already in the 1994 paper is that monospermic polyploid embryos are usually slowly developing ones with some cytoplasmic but not karyoplasmic arrest, with those cells arrested still continuing to duplicate or more its genome, creating complex mosaics. The haploid embryos usually are partegenetic lacking a well-formed spindle and thus divide a single chromosome set at random. 

We do not argue that 69XXX embryos may be sometimes purely triploid, but there is a huge caveat. There are three or more potential sources of triploid embryos, one are polyspermic embryos, which are invariably complex chromosomally abnormal and selected against by embryologist because they have three pronuclei. The other source is second polar body retention after ICSI. Those could be perfectly polyploid, but again embryologists usually select them against since they present a three pronuclei and a single PB. The third are giant eggs (Munné 2007), which are diploid instead of haploid, and thus are also selected against by embryologist. The few that escape embryologist detection, might indeed by purely triploid. In our database that is 0.2% of embryos (n= 91,000).

We refer the authors to our previous paper by Bisignano *et al.* (2011) in which we argued that the finding of UPD is being exaggerated by the fact that most embryos reported with UPD had other chromosome abnormalities, indicative of complex abnormalities. The finding of UPD is relevant in the absence of another deadly chromosome abnormality; otherwise the classification of the embryo is of complex abnormal, mosaic, or other, not UPD. 

Error rates by FISH can be high or low depending on the lab. In our hands was 7% (Colls *et al.* 2007), all explained by mosaicism. The authors group has yet to present data on cell by cell reanalysis of non-replaced embryos avoiding the question of mosaicism. Even the best diagnosis cannot get around day-3 mosaicism and achieve an error rate below 2-3%, measured by full reanalysis of not replaced embryos. Using FISH to analyze all the other cells of previously analyzed abnormal embryos has a very low error rate since many cells are analyzed and compared to each other, and is a cheap way to validate a technique, but any other technique can be used to validate another one, provided that all the cells of the non-replaced embryo are analyzed to differentiate mosaicism from technical error, something not published yet by the corresponding authors of the letter.

Sincerely,

## References

[1] Bisignano A, Wells D, Harton G, Munné S (2011). Preimplantation Ge- netic Diagnosis and Aneuploidy Screening for 24 chromosomes advantages and disadvantages of competing platforms. Reprod Biomed Online.

[2] Colls P, Escudero T, Zheng X, Lenzi M, Cinnioglu C, Cohen J, Munné S (2007). Increased efficiency of preimplantation genetic diagnosis for infertility through reanalysis of dubious signals. Fertil Steril.

[3] Colls P, Escudero T, Fischer J, Cekleniak N, Ben-Ozer S, Meyer B, Damien M, Grifo J, Hershlag A, Munné S (0000). Validation of array comparative genome hybridization for diagnosis of translocations in preimplantation human embryos. Reprod Biomed online.

[4] Fiorentino F, Kokkali G, Biricik A, Stavrou D, Ismailoglu B, De Palma R, Arizzi L, Harton G, Sessa M, Pantos K (2010). Polymerase chain reaction-based detection of chromosomal imbalances on embryos: the evolution of preimplantation genetic diagnosis for chromosomal translocations. Fertil. Steril.

[5] Munné S, Alikani M, Grifo J, Cohen J (1994). Monospermic polyploidy and atypical embryo morphology. Human Reprod.

[6] Munné S, Cohen J, and Elder K (2007). Chromosomal status of human embryos. In: Human Preimplantation Embryo Selection.
